# Polygenic Innate Immunity Score to Predict the Risk of Cytomegalovirus Infection in CMV D+/R- Transplant Recipients. A Prospective Multicenter Cohort Study

**DOI:** 10.3389/fimmu.2022.897912

**Published:** 2022-08-09

**Authors:** Marta Bodro, Carlos Cervera, Laura Linares, Belén Suárez, Jaume Llopis, Gemma Sanclemente, Sergi Casadó-Llombart, Mario Fernández-Ruiz, María Carmen Fariñas, Sara Cantisan, Miguel Montejo, Elisa Cordero, Isabel Oriol, María Angeles Marcos, Francisco Lozano, Asunción Moreno, GESITRA-IC/SEIMC/REIPI investigators

**Affiliations:** ^1^Infectious Diseases Department, Hospital Clinic de Barcelona - Institut d' Investigacions Biomèdiques August Pi i Sunyer (IDIBAPS), University of Barcelona, Barcelona, Spain; ^2^Department of Medicine, University of Alberta, Edmonton, AB, Canada; ^3^Immunology Department, Biomedical Diagnostic Center, Hospital Clínic de Barcelona, Barcelona, Spain; ^4^Immunoreceptors of the Innate and Adaptive Sistems, Institut d’Investigacions Biomèdiques August Pi i Sunyer (IDIBAPS), Barcelona, Spain; ^5^Hospital Universitario “12 de Octubre”, Instituto de Investigación Hospital “12 de Octubre” (imas12), Madrid, Spain; ^6^University Hospital “Marqués de Valdecilla”, Instituto de Investigación “Marqués de Valdecilla” (IDIVAL), University of Cantabria, Santander, Spain; ^7^Reina Sofía University Hospital, University of Cordoba, Cordoba, Spain; ^8^Hospital Universitario Cruces, Barakaldo, Universidad del País Vasco, Bilbao, Spain; ^9^Unit of Infectious Diseases, Microbiology, and Preventive Medicine, Virgen del Rocío University Hospital, Seville, Spain; ^10^Institute of Biomedicine of Seville (IBiS), Virgen del Rocío and Virgen Macarena University Hospitals/CSIC/University of Seville, Seville, Spain; ^11^Department of Medicine, University of Seville, Seville, Spain; ^12^University Hospital, Bellvitge Biomedical Research Institute (IDIBELL), L’Hospitalet de Llobregat, Barcelona, Spain; ^13^Biomedicine Department, University of Barcelona, Barcelona, Spain

**Keywords:** cytomegalovirus, solid organ transplantation, infectious disease, innate immunity, multicenter study

## Abstract

Several genetic polymorphisms of the innate immune system have been described to increase the risk of cytomegalovirus (CMV) infection in transplant patients. The aim of this study was to assess the impact of a polygenic score to predict CMV infection and disease in high risk CMV transplant recipients (heart, liver, kidney or pancreas). On hundred and sixteen CMV-seronegative recipients of grafts from CMV-seropositive donors undergoing heart, liver, and kidney or pancreas transplantation from 7 centres were prospectively included for this purpose during a 2-year period. All recipients received 100-day prophylaxis with valganciclovir. CMV infection occurred in 61 patients (53%) at 163 median days from transplant, 33 asymptomatic replication (28%) and 28 CMV disease (24%). Eleven patients (9%) had recurrent CMV infection. Clinically and/or functionally relevant single nucleotide polymorphisms (SNPs) from *TLR2*, *TLR3*, *TLR4*, *TLR7*, *TLR9*, *AIM2*, *MBL2*, *IL28*, IFI16, *MYD88*, *IRAK2* and *IRAK4* were assessed by real time polymerase chain reaction (RT-PCR) or sequence-based typing (PCR-SBT). A polygenic score including the *TLR4* (rs4986790/rs4986791), *TLR9* (rs3775291), *TLR3* (rs3775296), *AIM2* (rs855873), *TLR7* (rs179008), *MBL* (OO/OA/XAO), *IFNL3/IL28B* (rs12979860) and *IFI16* (rs6940) SNPs was built based on the risk of CMV infection and disease. The CMV score predicted the risk of CMV disease with an AUC of the model of 0.68, with sensitivity and specificity of 64.3 and 71.6%, respectively. Even though further studies are needed to validate this score, its use would represent an effective model to develop more robust scores predicting the risk of CMV disease in donor/recipient mismatch (D+/R-) transplant recipients.

## Introduction

Cytomegalovirus (CMV) is the most common opportunistic pathogen in solid organ transplant (SOT) recipients. While CMV infection can occur through reactivation of latent virus in seropositive recipients, CMV transmission from the donor is the riskiest clinical scenario in CMV seronegative transplant recipients because of the lack of a pre-existing CMV-specific immunity. CMV infection develops in 36% to 100% of SOT recipients, with rates of symptomatic disease between 11%-72% without prophylaxis (1, 2). In the case of donor/recipient CMV mismatch (D+/R-), more than 50% would develop CMV disease if no antiviral prophylaxis is administered (2). Some strategies have been developed in recent years in order to prevent CMV infection and disease such as extending antiviral prophylaxis from 3 to 6 months (3), using regimens containing mTOR inhibitors (4) or measuring CMV-specific T-cells immunity (5). However, CMV infection is still worrisome in SOT recipients and new biomarkers are necessary (6).

Immunosuppression predominantly impairs the adaptive immune response by blocking lymphocyte activation signalling pathways, depleting lymphocytes, or diverting lymphocyte trafficking (7). With the use of pharmacologic immunosuppression, the innate immune responses are critical in the defence against infection (8). Innate immune system components of either secreted (e.g., type I interferons, IFNs), membrane-bound (e.g., toll-like receptors; TLRs, mannose-binding lectin; MBL) or cytoplasmic (e.g., DNA cytoplasmic sensors) nature, are responsible for sensing and containment of the viral infection during the lag phase needed for adaptive immunity to become operative (9, 10). In the case of CMV, a large number of studies have reported the effect of single nucleotide polymorphisms (SNPs) in a variety of innate immune component genes (with especial relevance of *TLR4* and *MBL2*) on the risk of CMV infection (11–13). In the setting of organ transplant, donor and/or recipients genes encoding pattern recognition molecules and receptors such as *TLR (TLR2, 4 and 9)* or *MLB (MBL2)*, interferon (*IFNL3/IL28B*), cytokines (*IL12*, *IL10*), cytoplasmic sensors (*AIM2*, *IFI16, IRAK2, IRAK4*) and immune regulatory membrane-associated proteins (human programmed death-1, *PD1*) have been linked with an increased risk of CMV infection and disease among different transplant populations either solid organ or allogenic stem cell transplant patients (14–25).

TLR2, TLR3, TLR4 and TLR9 are pattern recognition receptors with a key role in innate immunity against viral infections. Accordingly, TLR genetic polymorphisms have been reported to impact the course of CMV infection. The heterozygous *TLR3* rs3775296 genotype is overrepresented in Polish children with CMV infection compared with uninfected cases (26). Likewise, *TLR9* variants (T/C, rs187084; C/T, rs352140) are associated with CMV disease in children (27) and *TLR2* variants (A>G, rs5743708) are associated with increased risk of congenital HCMV infection in a Polish cohort (28).

Although TLR4 is not directly involved in the recognition of CMV, TLR4 stimulation increases the number of CMV-specific CD4^+^ and CD8^+^ T cells by enhancing antigen presentation by dendritic cells (29). *TLR4* polymorphisms (rs4986790 and rs4986791) were associated with higher risk of CMV disease in kidney transplant recipient cohorts (30, 31).

TLR7 is also involved in viral recognition including CMV (29, 32), and it induces different IFN types such as IFN-λ3/IL-28B (33, 34). In fact, the rs12979860^T^ allele of *IFNL3IL28B* has a protective effect against CMV infection in allogeneic stem cell transplant patients (35). However, studies evaluating the impact of *TLR7* polymorphism on CMV infection risk are still pending.

*MyD88*, *IRAK2* and *IRAK4* are key downstream mediators of TLR signalling and are involved in fast CMV infection responses (36, 37). The *IRAK2* hypofunctional Leu392Val (rs3844283) variant has been associated with reduced spontaneous viral clearance (38).

Other receptors are involved in CMV recognition, including MBL, which recognizes glycoproteins present in the envelope of the virion. Hence, *MBL2* genetic variants that lead to low-MBL levels were associated with an increased risk of CMV infection and disease (31, 39).

Recognition of cytoplasmic DNA is also a remarkable function in the initiation of innate immune responses. Absent in melanoma 2 (*AIM2*) and *IFI16* are cytoplasmic sensors serving this role, and are involved in the control of CMV infection (40–43).

On this basis, we aimed to explore the impact of innate immunity on the risk of CMV infection and disease in patients at high risk to develop CMV infection such as CMV-seronegative recipients that received a CMV-seropositive donor in a prospective cohort of solid organ transplant recipients. To that end, we investigated SNPs from innate immune genes critically involved in eliciting effective anti-viral immune responses (*IFNL3/IL28B*, *MBL2*, *TLR2, TLR3, TLR4, TLR7, TLR9*, *MYD88*, *IRAK2*, *IRAK4, AIM2*, *IFI16*) to further explore their individual or combined potential impact in the risk of CMV infection.

## Material and Methods

### Setting and Study Population

We conducted a multicenter prospective observational study in seven Spanish hospitals (Hospital Clínic de Barcelona, Hospital de Bellvitge, Hospital Marqués de Valdecilla, Hospital 12 de Octubre, Hospital de Cruces, Hospital Virgen del Rocio and Hospital Reina Sofía) with an active transplant program. All adult CMV mismatch transplant recipients (CMV-seronegative recipients of grafts from CMV-seropositive donors) that signed informed consent document and agreed to participate in the study were prospectively included. Exclusion criteria were not to meet the inclusion criteria previously described and not to sign informed consent document. All patients were followed for 12 months post-transplant. Demographic data, type of transplant, immunosuppressive regimens, occurrence of biopsy-proven acute allograft rejection and infection episodes were prospectively recorded. The study was approved by each participating hospital research ethic committee. All patients signed informed consent at inclusion.

### Definitions

CMV DNAemia was defined as a positive CMV PCR in plasma without clinical symptoms. Symptomatic CMV infection was categorized as CMV disease and defined as CMV “viral syndrome” or tissue invasive disease according to published guidelines (44). CMV “viral syndrome” required the following criteria: (1) positive DNAemia for CMV; (2) a temperature > 38°C with no other accountable source; and (3) a leukocyte count < 4000/mm (3), an atypical lymphocyte concentration >3%, an elevation of transaminases or a platelet count <100,000/mm (3). A diagnosis of tissue-invasive disease required histopathological evidence of CMV (identification of inclusion bodies or viral antigens in biopsy material or bronchoalveolar lavage specimen cells by immunocytochemistry) with or without a positive PCR of CMV in the tissue. Late-onset CMV disease was defined as CMV disease occurring after prophylaxis completion.

Any CMV viral load by PCR without any previous positive CMV viral load post transplantation was considered primary CMV infection. A positive CMV PCR of 1000 IU/mL or higher after a primary infection with confirmed clearance (negative CMV viral load) was considered significant. Cytomegalovirus viral load was measured by quantitative real time polymerase chain reaction (qPCR) Cobas^®^ CMV (Roche^®^, Switzerland) according to the manufacturer’s instructions. The test can quantify CMV DNA over the range of 34.5 - 1E+07 IU/mL.

### Transplant Infection Prophylaxis Protocol

SOT patients received perioperative antibacterial prophylaxis for up to 48h after transplantation depending on each center’s protocol. During the study period, *Pneumocystis jirovecii* prophylaxis with trimethoprim-sulfametoxazole (one double strength tablet once three times a week) was given in the first six months after transplantation. All patients received prophylaxis with oral valganciclovir (900 mg once daily or dose adjusted by kidney function if there was renal impairment) or intravenous ganciclovir (5 mg/kg daily or dose adjusted by kidney function) for 100 days according to international guidelines (44). After antiviral prophylaxis completion, a preemptive strategy was applied (surveillance after prophylaxis) (44). A CMV PCR was performed every 15 days in the first month and then monthly for 12 months. Anti-fungal prophylaxis was given in high-risk recipients according to the American Society of Transplantation Infectious diseases Community of practice recommendations (45).

### Immunogenetic Analyses

Genomic DNA was extracted from a 1.5 mL whole blood sample using the QIAmp DNA blood mini nucleic acid extraction kit (QIAGEN GmbH, Hilden, Germany) following the manufacturer’s instructions, and stored at -80°C until use.

Genotyping of MBL2 was done by a polymerase chain reaction and sequence‐based typing (PCR160 SBT) technique as previously reported (46). Briefly, six single nucleotide polymorphisms (SNPs) in 161 the promoter region (-550 G>C, [H>L]; -221 C>G, [X>L]; +4 C>T, [P>Q]), and exon 1 (codon 52 CGT [Leu]>TGT [Arg], [A>D]; codon 54 GGC [Gly]>GAC [Asp], [A>B]; codon 57 GGA [Gly]>GAA [Glu], [A>C]) of the *MBL2* gene were analyzed. Variants at codons 52 (Arg; D), 54 (Asp; B) and 57 (Glu; C), are major determinants of low serum MBL levels (47) and are collectively named O, while A indicates the wild-type variants. SNPs at positions –550, −221 and +4 also influence serum MBL levels in individuals with the A variant. However, the functional effects of [H>L] and [P>Q] SNPs appear to be minor compared to [X>L], with X being the allele associated with lower *MBL2* expression. Accordingly, individuals were genotypically classified as high- (A/A, A/XA), intermediate- (XA/XA, A/O) or low- (XA/O, O/O) MBL producers.

Genotyping of SNPs in the TLR2 (rs5743708, CGG[Arg753]>CAG[Gln]), TLR3 (rs3775296, intron 1 + 95 C>A; rs3775291, CTC[Leu412]>TTC[Phe]), TLR4 (rs4986790, GAT[Asp299]>GGT[Gly]; rs4986791, ACC[Thr399]>ATC[Ile]), TLR7 (rs179008, CAA[Gln11]>CTA[Leu]), TLR9 (rs5743836, 5’UTR -1486 T>C; rs187084, 5’UTR -1237 T>C; rs352140, CCG[Pro545]>CCA[Pro]), MyD88 (rs6853, 3’UTR A>G), IRAK2 (rs3844283, CTG[Leu392]>GTG[Val]), IRAK4 (rs4251513, intron G>C), AIM2 (rs855873, intron G>A) IFI16 (rs6940, ACT[Thr723]>TCT[Ser]) and IFNL3/IL28B (rs12979860, intron C>T) genes were performed by allelic discrimination using TaqMan SNP-genotyping Assays (Applied Biosystems/Thermo Fisher Scientific, Waltham, MA, USA) on a LightCycler 480 Instrument II (Roche) according to manufacturer’s instructions. Allelic frequencies for the autosomal SNPs genotyped are shown in **Supplementary Table S3**. All of them were in Hardy-Weinberg equilibrium except for the *IRAK4* rs4251513 SNP.

### Statistical Methods

Statistical tests were performed using SPSS Version 19 (SPSS, Chicago, IL). For comparisons of study groups, two-sided Mann–Whitney U-Test for nonparametric independent samples was used. Clinical and infection-specific characteristics were compared across groups using Fisher’s exact test or χ (2) test for categorical variables, and Student’s t-test for continuous variables. Two-sided p-values <0.05 were considered statistical significant.

The CMV genetic risk score was constructed using a logistic regression model with all independent variables. The coefficients of the independent variables with confidence intervals of 80% not including zero were extracted and a score was obtained including these selected variables depending on the coefficient weight. The discriminatory power of the score was evaluated by the area under the receiver operating characteristics (ROC) curve and the 95% confidence interval (CI). Then, a cut-off value to estimate the diagnostic sensitivity and specificity in the validation set was selected.

The sample size of the study was calculated accounting an estimate 40% incidence of late CMV infection in patients with any genetic polymorphism compared to 15% of patients with a wild type genotype. From published data, 10% of transplant subjects will have a genetic polymorphism. To demonstrate the aforementioned risk difference with an alpha risk of 5% and a power of 80%, we needed to include 129 patients with a solid organ transplant and a D +/R- serological pattern.

## Results

During the 2-year recruitment period (2013-2015), 116 CMV mismatch (D+/R-) transplant recipients from 7 Spanish hospitals were included. **Table 1** shows the baseline, demographic and clinical characteristics of enrolled patients at inclusion. Sixty-one patients (53%) had at least one episode of CMV infection, 33 of them (28%) clinically asymptomatic and 28 categorized as CMV disease (24%), none of them occurring while on antiviral prophylaxis. Eleven patients (9%) had recurrent CMV infection. Median days from transplantation to CMV infection was 163 (SD 73). Forty-nine patients (42%) presented at least one episode of bacterial infection during the study period, 8 (7%) non-CMV viral infections and 7 (6%) invasive fungal infections.

**Table 1:** Baseline, demographic and clinical characteristics of patients.

Variable	n=116
Age (median, SD)	49 ±14.1
Gender, male, n(%)	93 (80)
Days of follow-up (median, SD)	684 (497)
Type of transplantation, n(%)	
◾ Kidney	66 (57)
◾ Liver	34 (29)
◾ Heart	10 (9)
◾ Multivisceral transplantation	6 (5)
Induction immunosuppressive treatment, n(%)	
◾ None	39 (34)
◾ Lymphocyte-depleting antibody	45 (39)
◾ Anti-thymocyte globulin	31 (28)
Maintenance immunosupressive treatment, n(%)	
◾ Calcineurin inhibitors + mycophenolate mofetil +prednisone	96 (83)
◾ Calcineurin inhibitors + mTOR inhibitors+prednisone	18 (15)
◾ mTOR inhibitors + mycophenolate mofetil + prednisone	1 (1)
Postransplantation non- infectious complications, n(%)	
◾ Acute rejection (only biopsy proven)	16 (14)
◾ Hemodialysis	22 (19)
◾ Surgical reintervention related to transplantation	22 (19)
◾ Graft loss	3 (2)
◾ Death	1 (1)
**Cytomegalovirus infection**, n(%)	61 (53)
First episode	
Asymptomatic replication	33 (28)
CMV disease	28 (24)
CMV syndrome	11 (10)
End-organ CMV disease	17 (15)
Median days post transplantation (SD)	163 (73)
Viral load (median, SD)	66351(188736)
Second episode	11 (10)
Asymptomatic replication	9 (8)
CMV disease	2 (2)
CMV syndrome	1 (1)
End-organ CMV disease	1 (1)
Median days post transplantation (SD)	239 (72)
Viral load (median, SD)	71647 (175673)
Third episode	
Asymptomatic replication	4 (3)
Median days post transplantation (SD)	289 (42)
Viral load (median, SD)	52354 (61217)
Bacterial infection, n(%)	49 (42)
Other viral infections , n(%)	8 (7)
Invasive fungal infection, n(%)	7 (6)

**Table 2** shows the distribution of *TLR2, TLR3, TLR4, TLR7, TLR9, AIM2, MBL2, IFI16, IFNL3/IL28B, MYD88, IRAK2 and IRAK4* genotypes by group. No differences in CMV infection, either asymptomatic CMV infection or disease, was found between groups. Nevertheless, we performed a subanalysis according to the type of organ transplanted (**Supplementary Tables S1**, **S2**) and found that kidney and liver recipients presenting with *TLR4* rs4986790/rs4986791 polymorphism presented more frequently CMV infection comparing with those with wild type (70% *vs* 41% and 100% *vs* 51% respectively, p=0.05). Additionally, *IFNL3/IL28B* rs12979860 polymorphism was considered a protector factor against CMV infection in liver recipients (27% *vs* 74%, p=0.03).

**Table 2:** Univariate analysis of CMV infection according to TLR 2, 3, 4, 7, 9, AIM2, MBL2, IFI16, IL28B, MYD88, IRAK2 and 4 genotypes.

SNP genotype	Asymptomatic CMV infection	p	CMV disease		p	CMV infection	p
			Viral syndrome	Tissue-invasivedisease			
***TLR2* rs5743708**							
Wild type GG (n=111)	31 (28%)	0.6	10 (9%)	17 (15%)	0.5	58 (52%)	0.5
Variant GA (n=5)	2 (40%)		1 (20%)	0		3 (60%)	
***TLR3* rs3775296**							
Wild type CC (n=74)	22 (30%)	0.4	7 (9%)	9 (12%)	0.3	38 (38%)	0.4
Variant AA or AC (n=42)	11(33%)		4 (9%)	8 (19%)		23 (55%)	
Homozygous AA (n=2)	0	0.2	0	0		0	0.9
Heterozygous AC (n=40)	11(27%)		4 (10%)	8(20%)		23 (57%)	
***TLR3* rs3775291**							
Wild type CC (n=51)	15 (29%)	0.5	4 (8%)	7 (13%)	0.8	26 (51%)	0.4
Variant TT or CT (n=65)	18 (28%)		7 (11%)	10 (15%)		35 (54%)	
Homozygous TT (n=18)	6 (33%)	0.9	1 (6%)	2 (11%)		9 (50%)	0.9
Heterozygous CT (n=67)	12 (18%)		6 (9%)	8 (12%)		26 (39%)	
***TLR4* rs4986790 / rs4986791**							
Wild type AA / CC (n=97)	28 (29%)	0.3	8 (8%)	13 (13%)	0.6	49 (50%)	0.3
Variant CC or AC / TT or CT (n=19)	5 (26%)		3 (16%)	4 (21%)		12 (63%)	
Homozygous CC / TT (n=2)	0	0.4	1 (50%)	0	0.3	1 (50%)	0.5
Heterozygous AC / CT (n=17)	5 (29%)		2 (12%)	4 (24%)		11 (65%)	
***TLR7* ex3 rs179008**							
Wild type AA (n=87)	25 (29%)	0.3	7 (8%)	12 (14%)	0.7	44 (51%)	0.4
Variant AT or TT (n=29)	8 (28%)		4 (14%)	5 (17%)		17 (59%)	
Homozygous TT (n=26)	7 (27%)	0.7	4 (15%)	3 (12%)	0.4	14 (54%)	0.2
Heterozygous AT (n=3)	1 (33%)		0	2 (67%)		3 (100%)	
***TLR9* ex4 rs3775291**							
Wild type AA (n=33)	9 (27%)	0.3	6 (18%)	4 (12%)	0.2	19 (57%)	0.3
Variant AG or GG (n=83)	24 (29%)		5 (6%)	13 (16%)		42 (51%)	
Homozygous GG (n=22)	6 (27%)	0.6	3 (14%)	3 (14%)	0.2	12 (55%)	0.7
Heterozygous AG (n=61)	18 (30%)		2 (3%)	10 (16%)		30 (49%)	
***AIM2* rs855873**							
Wild type GG (n=98)	30 (31%)	0.3	9 (9%)	14 (14%)	0.6	53 (54%)	0.3
Variant AG-AA (n=18)	3 (17%)		2 (11%)	3 (17%)		8 (44%)	
***MBL2* ex1**							
High A/A or XA/A (n=65)	16 (25%)	0.9	5 (8%)	12 (25%)	0.2	33 (51%)	0.9
Intermediate A/0 or XA/XA (n=36)	13 (36%)		3 (8%)	2 (6%)		18 (50%)	
Low 0/0 or XA/0 (n=15)	4 (27%)		3 (20%)	3 (20%)		10 (66%)	
***IFI16* rs6940**							
Wild type AA (n=98)	26 (27%)	0.2	10 (10%)	15 (15%)	0.5	51 (52%)	0.5
Variant AT or TT (n=18)	7 (39%)		1 (6%)	2 (11%)		10 (55%)	
***IL28* rs12979860**							
Wild type CC (n=64)	17 (27%)	0.5	7 (11%)	10 (16%)	0.4	34 (53%)	0.5
Variant CT or TT (n=52)	16 (31%)		4 (8%)	7 (13%)		27 (52%)	
Homocygous TT (n=8)	4 (9%)	0.7	–	–	0.4	4 (50%)	1
Heterozygous CT (n=44)	12 (27%)		4 (9%)	7 (16%)		23 (52%)	
***MYD88* rs6853**							
Wild type AA (n=82)	25 (30%)	0.5	9 (11%)	11 (13%)	0.6	45 (55%)	0.3
Variant GG or AG (n=34)	8 (24%)		2 (6%)	6 (18%)		16 (47%)	
Homozygous GG (n=2)	–	0.5	–	1 (50%)	0.5	1 (50%)	0.7
Heterozygous AG (n=32)	8 (25%)		2 (6%)	5 (16%)		15 (47%)	
***IRAK2* rs3844283**							
Wild type CC (n=50)	16 (32%)	0.3	6 (12%)	4 (8%)	0.2	26 (52%)	0.9
Variant GG or CG (n=66)	17 (26%)		5 (8%)	13 (20%)		35 (53%)	
Homozygous GG (n=9)	2 (22%)	0.6	–	2 (22%)	0.4	4 (44%)	0.7
Heterozygous CG (n=57)	15 (26%)		5 (9%)	11 (19%)		31 (54%)	
***IRAK4* rs4251513**							
Wild type CC (n=38)	13 (34%)	0.8	3 (8%)	7 (18%)	0.7	23 (60%)	0.2
Variant GG or CG (n=78)	20 (26%)		8 (10%)	10 (13%)		48 (61%)	
Homozygous GG (n=34)	11 (32%)	0.4	4 (12%)	5 (15%)	0.7	20 (59%)	0.7
Heterozygous CG (n=44)	9 (20%)		4 (9%)	5 (11%)		18 (41%)	

The CMV polygenic score was built through a stepwise elimination process (**Table 3**). The final calculated score is described by the following formula: Genetic risk score for CMV disease = 0.68 x *TLR4* rs4986790/rs4986791 – 0.56 x *TLR9* rs3775291 + 0.34 x *TLR3* rs3775296+ 0.47 x *AIM2* rs855873+ 0.71 x *TLR7* rs179008-0.43 x *MBL OO/OA/XAO* – 0.49 x *IFNL3/IL28B* rs12979860 – 0.55 x *IFI16* rs6940.

**Table 3:** Calculation of the CMV genetic risk score: variables selected in the logistic regression model.

Variable	Coefficient (β)	Standard error	Wald χ^2^	p value
***TLR3* rs3775296**	0.34	0.22		
***TLR4* rs4986790/rs4986791**	0.68	0.32		
***TLR7* rs179008**	0.71	0.39		
***TLR9* rs3775291**	0.56	0.30		
***AIM2* rs855873**	0.47	0.25		
***MBL2 (O/O, O/A, XA/O)***	0.43	0.24		
***IFI16* rs6940**	-0.55	0.28		
***IFNL3/IL28* rs12979860**	-0.49	0.25		
			8.2	0.004

**Figure 1:**
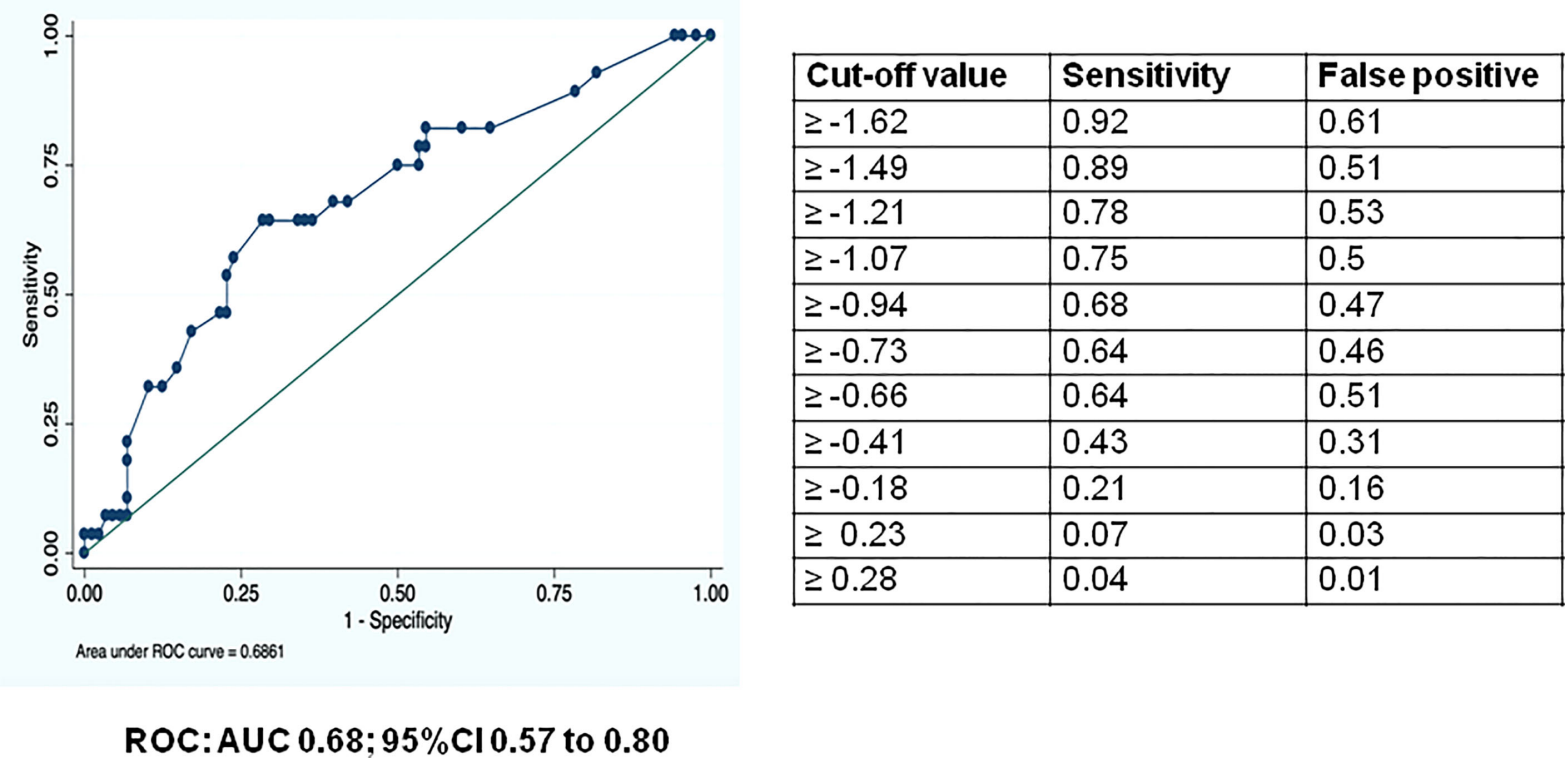
Receiver operating characteristics (ROC) curve and area under the ROC curve (AUC) for assessing the discriminatory power of the risk score model for the prediction of CMV disease in SOT recipients, cut-off values for the ROC curve. ROC: AUC 0.68; 95%CI 0.57 to 0.80.

The discriminatory power of the score was assessed by the area under the receiver operation characteristics (ROC) curve and area under the ROC curve (AUCs). The discriminatory power of the CMV polygenic risk score and cut-off values for the ROC curve are shown in **Figure 1**. The optimal estimated cut-off value of the score could be established in -0.36, conferring a sensitivity of the score of 62.3% (CI 53.5-71.1), a specificity of 52.73% (43.6-61.8), a positive predictive value of 0.59 (CI 0.5-0.7) and a negative predictive value of 0.56 (CI 0.4-0.6). Nevertheless, in order to improve specificity, the cut-off of 0 confers a sensitivity of the score of 43% (CI 33.6-51.6), a specificity of 71% (CI 62.6-79.2), a positive predictive value of 0.62 (CI 0.5-0.7) and a negative predictive value of 0.53 (0.4-0.6).

The values of sensitivity, specificity, positive and negative predictive values of the score are shown in **Table 4**. *TLR4* SNPs rs4986790/rs4986791 had the highest predictive value among these parameters, especially in terms of specificity (87.3%).

**Table 4:** Area under the ROC curve (AUCs) of innate immune receptor gene SNPs for the prediction of CMV disease patients in SOT recipients with sensitivity and specificity.

Gene SNP	AUCs (95%CI)	SEN %	SPE %	PPV %	NPV %	LR+	LR-
***TLR3* 3775296**	0.51 (0.42, 0.61)	37.7	65.4	54.8	48.6	1.09	0.95
***TLR4* rs4986790/rs4986791**	0.53 (0.44, 0.63)	19.7	87.3	63.2	49.5	1.54	0.92
***TLR7* rs179008**	0.53 (0.44, 0.63)	27.9	78.2	58.6	49.4	1.27	0.92
***TLR9* rs3775291**	0.47 (0.38, 0.57)	68.8	25.4	50.6	42.4	0.92	1.22
***AIM2* rs855873**	0.47 (0.38, 0.56)	13.1	81.8	44.4	45.9	0.72	1.06
***MBL2* (O/O, O/A, XA/O)**	0.51 (0.44, 0.57)	16.4	85.4	56.6	48.0	1.13	0.98
***IFI16* rs6940**	0.51 (0.41, 0.60)	16.4	85.4	55.6	48.0	1.13	0.98
***IFNL3/IL28* rs12979860**	0.49 (0.40, 0.58)	44.2	54.5	51.9	46.9	0.97	1.02

*ROC receiver operation characteristics, SEN sensitivity, SPE specificity, PPV positive predictive value, NPV negative predictive value, LR+ positive likelihood ratio, LR- negative likelihood ratio.

## Discussion

In this prospective study of a large multicenter cohort of SOT recipients at high risk to develop CMV infection and disease, we built a polygenic score based on innate immune receptors gene SNPs to predict the probability of developing post-transplant CMV infection and disease. Many previous studies have addressed the risk of CMV infection in transplant patients according to the innate immune single gene SNPs. Our score was based on these previous associations and SNPs in innate immune receptor genes with theoretical influence in the immune control of CMV replication.

Several individual TLRs are involved in sensing viral nucleic acids and structural components. *TLR3* recognizes viral double-stranded RNA (dsRNA) during human CMV replication and activates antiviral immune responses through production of type I IFN and inflammatory cytokines. Interestingly, increased frequency of the heterozygous *TLR3* rs3775296 genotype has been reported in Polish children with CMV infection compared with uninfected cases (26). In line with this, the same group also found association of *TLR9* variants (T/C, rs187084; C/T, rs352140) with CMV disease in children (27) and of *TLR2 (*A>G, rs5743708) with increased risk of congenital HCMV infection in Polish fetuses and neonates (28). *TLR4* is not directly involved in the recognition of CMV, but it has been demonstrated that *TLR4*-ligands enhance the ability of dendritic cells to present CMV antigens resulting in an increased number of antigen-specific activated CD4^+^ and CD8^+^ T cells (29). In our study *TLR4* polymorphism had a specificity of 87% to detect CMV disease and what is more, in the subgroup of kidney and liver recipients *TLR4* polymorphism was associated with CMV infection, in line with other studies performed in kidney transplant recipients’cohorts including non-only high risk patients (30, 31). In the same way, we found that *TLR3*, *TLR7* and *TLR9* polymorphisms increased the risk of CMV disease.

Recognition of cytoplasmic DNA is an important immunological signature that marks the initiation of innate immune response. Absent in melanoma 2 (*AIM2*) is a cytoplasmic sensor that recognizes DNA of microbial or host origin (40) and give rise to a multiprotein complex – the so called *AIM2* inflammasome - which plays a pivotal role in the host immune response to multiple pathogens. Compared to wild-type cells, *AIM2*-deficient macrophages showed a limited ability to activate caspase-1, process IL-1β, and induce cell death. In addition, *AIM2*-deficient cells were unable to efficiently control CMV infection, as the transcription of virus DNA polymerase gene UL54 and major tegument protein gene UL83 were higher compared to wild-type cells (42). *IFI16* is another cytoplasmic DNA sensor that acts as restriction factor for CMV replication (41). When *IFI16* undergoes phosphorylation relocalizes to the cytoplasm of CMV-infected cells and restricts CMV replication by downregulating viral mRNAs and their protein expression (43). Importantly, at the present study we found that both *AIM2* and *IFI16* polymorphisms were related with CMV disease.

MBL is a serum C-type lectin produced in the liver that binds carbohydrates present on a wide variety of bacterial, fungal, viral and parasitic surfaces (48). The CMV envelope is extremely complex and shares capsid glycoproteins with similar structural properties to other herpes virus. The gM and gN glycoproteins are the most important and abundant in the envelope of the virion, and the gM/gN complex formation is essential for maintaining virion infectivity. Furthermore, other glycoproteins such as gB and gH mediate viral entry into cells and are potential targets for MBL binding. So, genotypes responsible for low-MBL levels were associated with an increased risk of CMV infection and disease (31, 39), accordingly with our results.

*IFN-λ3/IL-28B* is a type III interferon that plays a role in the TLR-induced anti-viral activity (49). A protective effect of the rs12979860^T^ allele of the *IFNL3IL28B* against CMV infection in allogeneic stem cell transplant patients has been described (35), in agreement with the results of the present study.

Strategies for preventing CMV infection and disease in CMV mismatch patient are frequently debated in SOT recipients. Since the introduction of antiviral prophylaxis with i.v. ganciclovir or oral valganciclovir, a 100 to 200 day course of these drugs has been proposed for patients at high risk to develop CMV disease. However, this strategy is associated with high rates of toxicity (leucopoenia) leading to early discontinuation of prophylaxis. In addition, antiviral prophylaxis is associated with late-onset CMV disease and with an independently associated greater risk of mortality (50, 51). On the other hand, pre-emptive strategies have been historically associated with suboptimal results (52), although more recent data seems promising in liver transplant recipients (53). Furthermore, T cell immune functional assays such as ELISpot or QuantiFERON assays have shown association with higher incidence of CMV infection (5). Measurement of such parameters could potentially be used to inform the risk of infection as recommended by some international guidelines (44) although no interventional studies using these tests have been performed. Accordingly, there are some potential clinical scenarios where the CMV score could be used. Extending antiviral prophylaxis or using a longer pre-emptive strategy after antiviral prophylaxis could be considered in CMV-seronegative recipients of grafts from CMV-seropositive donors undergoing transplantation with high CMV score. Additionally, using mTOR inhibitors as maintenance immunosuppressive therapy due to protective anti CMV effect or using less intense immunosuppression are potential strategies for preventing CMV complication in patients at higher risk (4). Finally, this score can be a starting point for the creation of new scores to evaluate the risk of CMV infection and disease that include other parameters such as T cell immune functional assays and immunosuppressive regimen. Other proposal could be that this score can be used as a screening test and secondly, MBL levels and TLRs function could be measured by stimulation of the peripheral mononuclear cells with specific TLR-ligands in patients presenting with a CMV high-risk score to better assess the CMV risk.

The main strength of our study is the prospective inclusion of a large cohort of CMV mismatch transplant recipients from different centers with equivalent strategies of prophylaxis, which allowed obtaining granular data on immunosuppression and CMV events. Moreover, the genetic score predicts the risk of CMV disease using innate immune gene receptors polymorphisms. Nevertheless, an external validation of our results is mandatory in order to reaffirm our results.

Some caveats should be highlighted. Polygenic scores should be based on relevant genomic-wide associated studies (54). Our score was built based on previous published information analyzing single-gene SNPs in a variety of cohorts. Whole-genome sequencing studies to characterize the genetic profile associated to higher risk of CMV in transplant patients would require the coordination of a large international cohort. We propose a new line of research to apply precision medicine in predicting the management strategy of transplant patients at risk of CMV. The performance of our proposed polygenic score should be validated by other prospective cohorts. However, this pilot polygenic score could be improved by adding other candidate SNPs to the model. Furthermore, the heterogeneity of the cohort and the varying degree of CMV infection risk according to the type of allograft and immunosuppression represents another limitation to interpret the data. Certainly, building a model capable to control for all these variables would require a much larger cohort. Finally, other limitation of our study is that we performed the MBL genotyping only in the recipient. Taking into account that this protein is synthesized by the liver, MBL genotyping should have been performed in the donor in liver recipients.

To conclude, we propose a CMV polygenic score to predict the risk of CMV disease in CMV D+/R-, based on *TLR4* (rs4986790/rs4986791), *TLR9* (rs3775291), *TLR3* (rs3775296), *AIM2* (rs855873), *TLR7* (rs179008), *MBL* (OO/OA/XAO), *IFNL3/IL28B* (rs12979860) and *IFI16* (rs6940) genetic variants. Our score may help to identify patients at low risk of developing CMV disease that may benefit of shortening antiviral prophylaxis. On the other hand, those patients at high genetic risk of CMV may benefit of prolonged prophylaxis or even lifelong prophylaxis depending on the type of transplant. Further studies to validate the use of polygenic scores to predict the risk of CMV infection in SOT recipients are needed.

## Data Availability Statement

The original contributions presented in the study are included in the article/**Supplementary Material**, further inquiries can be directed to the corresponding author/s.

## Ethics Statement

The studies involving human participants were reviewed and approved by Comitè d'Ètica de la Investigació amb medicaments (CEIm), Hospital Clinic de Barcelona (2012/7530). The patients/participants provided their written informed consent to participate in this study.

## Author Contributions

MB, CC, FL and AM participated in research design. MB, CC and FL participated in the writing of the paper. MB, CC, LL, BS, JL, GS, MF-R, MF, SC, MM, EC, IO, MAM, FL SCL and AM participated in the performance of the research. MB, CC and JL contributed in analytic tools and data analysis. All authors contributed to the article and approved the submitted version.

## Funding

This work was supported by Spanish Ministerio de Sanidad y Consumo (FIS PI12/01743), Instituto de Salud Carlos III, Fondo Europeo de Desarrollo Regional (FEDER). Unión Europea. “Una manera de hacer Europa”; Network for the Research in Infectious Diseases (REIPI) from the Instituto de Salud Carlos III, Madrid, Spain; and Ministerio de Ciencia e Innovación (PID2019-106658RB-I00, funded by MCIN/AEI/10.13039/501100011033). MF-R holds a research contract “Miguel Servet” (CP18/00073) from the Instituto de Salud Carlos III, Spanish Ministerio de Ciencia e Innovación.

## Conflict of Interest

FL is founder and *ad honorem* scientific advisor or Sepsia Therapeutics S.L.

The remaining authors declare that the research was conducted in the absence of any commercial or financial relationships that could be construed as a potential conflict of interest.

## Publisher’s Note

All claims expressed in this article are solely those of the authors and do not necessarily represent those of their affiliated organizations, or those of the publisher, the editors and the reviewers. Any product that may be evaluated in this article, or claim that may be made by its manufacturer, is not guaranteed or endorsed by the publisher.

## Appendix I: Members of the GESITRA-IC/SEIMC/REIPI investigators related to the study:

JM Aguado, Hospital Universitario “12 de Octubre”, Instituto de Investigación Hospital “12 de Octubre” (imas12) Madrid, Spain

J.Torre-Cisneros, Reina Sofía University Hospital/University of Cordoba, Cordoba, Spain.

J. Carratala, University Hospital, Bellvitge Biomedical Research Institute (IDIBELL), L’Hospitalet de Llobregat, Barcelona, Spain

C. Lumbreras, Hospital Universitario “12 de Octubre”, Instituto de Investigación Hospital “12 de Octubre” (imas12) Madrid, Spain

M. Montejo. Hospital Universitario de Cruces. Bilbao, Spain

C. Roca, Department of Medicine, University of Seville, Virgen del Rocío University Hospital, Institute of Biomedicine of Seville (IBiS),/CSIC/University of Seville,

M. Gutierrez University Hospital “Marqués de Valdecilla”, Instituto de Investigación “Marqués de Valdecilla” (IDIVAL), University of Cantabria, Santander, Spain

C. Rico. University Hospital “Marqués de Valdecilla”, Instituto de Investigación “Marqués de Valdecilla” (IDIVAL), University of Cantabria, Santander, Spain

L. Muñoz. Hospital Clinic - IDIBAPS, University of Barcelona, Spain

A. Páez-Vega. Reina Sofía University Hospital/University of Cordoba, Cordoba, Spain.

E. Vidal Verdú. Reina Sofía University Hospital/University of Cordoba, Cordoba, Spain.

A.Rodríguez-Benot. Reina Sofía University Hospital/University of Cordoba, Cordoba, Spain.

J. Colmenero. Hospital Clinic - IDIBAPS, University of Barcelona, Spain

F. Cofan. Hospital Clinic - IDIBAPS, University of Barcelona, Spain

N. Armiger. Hospital Clinic - IDIBAPS, University of Barcelona, Spain

M. Cardona. Hospital Clinic - IDIBAPS, University of Barcelona, Spain

Y. Zboromyrska. Hospital Clinic - IDIBAPS, University of Barcelona, Spain
