# Supplementary material for: Polygenic Innate Immunity Score to Predict the Risk of Cytomegalovirus Infection in CMV D+/R- Transplant Recipients. A Prospective Multicenter Cohort Study
Source: Front Immunol. 2022 Aug 9;13:897912. doi: 10.3389/fimmu.2022.897912 (PMC9397545; doi:10.3389/fimmu.2022.897912)
Supplement: Supplementary file 1 [file Table_1.docx]

**Supplementary Table S1. Univariate analysis of CMV infection according to *TLR2, 3, 4, 7, 9, AIM2, MBL2, IFI16, IFNL3/IL28B*, *MYD88, IRAK2* and *4* genotypes in kidney recipients.**

| **SNP genotype** | **Asymptomatic CMV infection** | **CMV disease**  Viral syndrome Tissue-invasive  disease | | **CMV infection** | **p** |
| --- | --- | --- | --- | --- | --- |
| ***TLR2* rs5743708**  Wild type GG (n=62)  Variant GA (n=4) | 15  1 | 5  1 | 10  0 | 30 (50%)  2 (50%) | 0.67 |
| ***TLR3* rs3775296**  Wild type CC (n=44)  Variant AA or AC (n=22)  Homozygous AA (n=1)  Heterozygous AC (n=21) | 10  6  0  6 | 4  2  0  2 | 6  3  1  2 | 20 (45%)  11 (50%)  1 (100%)  10(47%) | 0.5 |
| ***TLR3* rs3775291**  Wild type CC (n=32)  Variant TT or CT (n=34)  Homozygous TT (n=9)  Heterozygous CT (n=25) | 10  6  3  3 | 2  4  1  3 | 3  7  2  5 | 15 (47%)  17 (50%)  6 (66%)  11 (44%) | 0.5 |
| ***TLR4* rs4986790 / rs4986791**  Wild type AA / CC (n=56)  Variant CC or AC / TT or CT (n=10)  Homozygous CC / TT (n=2)  Heterozygous AC / CT (n=8) | 14  2  0  2 | 5  1  1  0 | 4  3  0  3 | 23 (41%)  6 (60%)  1 (50%)  5 (62%) | 0.05 |
| ***TLR7* ex3 rs179008**  Wild type AA (n=48)  Variant AT or TT (n=18)  Homozygous TT (n=16)  Heterozygous AT (n=2) | 12  4  4  0 | 4  2  2  0 | 7  3  1  2 | 23 (48%)  9 (50%)  7 (43%)  2 (100%) | 0.32 |
| ***TLR9* rs5743836**  Wild type AA (n=17)  Variant AG or GG (n=49)  Homozygous GG (n=14)  Heterozygous AG (n=35) | 2  14  4  10 | 5  1  0  1 | 3  7  1  6 | 10 (59%)  22 (45%)  5 (36%)  17 (48%) | 0.44 |
| ***AIM2* rs855873**  Wild type GG (n=52)  Variant AG-AA (n=14) | 14  2 | 4  2 | 8  2 | 26 (50%)  6 (43%) | 0.43 |
| ***MBL2* ex1**  High A/A or XA/A (n=35)  Intermediate A/0 or XA/XA (n=18)  Low 0/0 or XA/0 (n=13) | 8  6  2 | 2  1  3 | 8  0  2 | 18 (51%)  7 (39%)  7 (54%) | 0.62 |
| ***IFI16* rs6940**  Wild type AA (n=61)  Variant AT or TT (n=5) | 15  1 | 6  0 | 9  1 | 30 (49%)  2 (40%) | 0.52 |
| ***IFNL3/IL28B* rs12979860**  Wild type CC (n=34)  Variant CT or TT (n=32)  Homocygous TT (n=5)  Heterozygous CT (n=27) | 7  9  2  7 | 3  3  0  3 | 5  5  0  5 | 15 (44%)  17 (53%)  2 (40%)  15 (55%) | 0.62 |
| ***MYD88* rs6853**  Wild type AA (n=45)  Variant GG or AG (n=21)  Homozygous GG (n=1)  Heterozygous AG (n=20) | 11  5  0  5 | 5  1  0  1 | 7  3  0  3 | 23 (51%)  9 (43%)  0  9 (45%) | 0.55 |
| ***IRAK2* rs3844283**  Wild type CC (n=29)  Variant GG or CG (n=37)  Homozygous GG (n=7)  Heterozygous CG (n=30) | 8  8  1  7 | 4  2  0  2 | 2  8  2  6 | 14 (48%)  18 (49%)  3 (43%)  15 (50%) | 0.94 |
| ***IRAK4* rs4251513**  Wild type CC (n=19)  Variant GG or CG (n=47)  Homozygous GG (n=20)  Heterozygous CG (n=27) | 4  12  6  6 | 2  4  1  3 | 3  7  4  3 | 9 (47%)  23 (49%)  11 (55%)  12 (44%) | 0.77 |
