# Supplementary material for: Polygenic Innate Immunity Score to Predict the Risk of Cytomegalovirus Infection in CMV D+/R- Transplant Recipients. A Prospective Multicenter Cohort Study
Source: Front Immunol. 2022 Aug 9;13:897912. doi: 10.3389/fimmu.2022.897912 (PMC9397545; doi:10.3389/fimmu.2022.897912)
Supplement: Supplementary file 2 [file Table_2.docx]

**Supplementary Table S2. Univariate analysis of CMV infection according to *TLR 2, 3, 4, 7, 9, AIM2, MBL2, IFI16, IFNL3/IL28B*, *MYD88, IRAK2* and *4* genotypes in liver recipients.**

| **SNP genotype** | **Asymptomatic CMV infection** | **CMV disease**  Viral syndrome Tissue-invasive  disease | | **CMV infection** | **p** |
| --- | --- | --- | --- | --- | --- |
| ***TLR2* rs5743708**  Wild type GG (n=34)  Variant GA (n=0) | 11 | 5 | 4 | 20 (59%) | - |
| ***TLR3* rs3775296**  Wild type CC (n=19)  Variant AA or AC (n=15)  Homozygous AA (n=0)  Heterozygous AC (n=15) | 7  4  0  4 | 3  2  0  2 | 1  3  0  3 | 11 (58%)  9 (60%)  0  9 (60%) | 0.59 |
| ***TLR3* rs3775291**  Wild type CC (n=15)  Variant TT or CT (n=19)  Homozygous TT (n=5)  Heterozygous CT (n=14) | 4  7  1  6 | 2  3  0  3 | 2  2  0  2 | 8 (53%)  12 (63%)  1 (20%)  11 (78%) | 0.06 |
| ***TLR4* rs4986790 / rs4986791**  Wild type AA / CC (n=29)  Variant CC or AC / TT or CT (n=5)  Homozygous CC / TT (n=0)  Heterozygous AC / CT (n=5) | 9  2  0  2 | 3  2  0  2 | 3  1  0  1 | 15 (52%)  5 (100%)  0  5 (100%) | 0.05 |
| ***TLR7* ex3 rs179008**  Wild type AA (n=27)  Variant AT or TT (n=7)  Homozygous TT (n=6)  Heterozygous AT (n=1) | 8  3  2  1 | 3  2  2  0 | 3  1  1  0 | 14 (52%)  6 (86%)  5 (83%)  1 (100%) | 0.25 |
| ***TLR9* rs5743836**  Wild type AA (n=13)  Variant AG or GG (n=21)  Homozygous GG (n=5)  Heterozygous AG (n=16) | 5  6  1  5 | 1  4  3  1 | 1  3  0  3 | 7 (54%)  13 (62%)  4 (80%)  9 (56%) | 0.57 |
| ***AIM2* rs855873**  Wild type GG (n=32)  Variant AG-AA (n=2) | 10  1 | 5  0 | 4  0 | 19 (59%)  1 (50%) | 0.66 |
| ***MBL2* ex1**  High A/A or XA/A (n=21)  Intermediate A/0 or XA/XA (n=11)  Low 0/0 or XA/0 (n=2) | 5  4  2 | 3  2  0 | 3  1  0 | 11 (52%)  7 (64%)  2 (100%) | 0.4 |
| ***IFI16* rs6940**  Wild type AA (n=25)  Variant AT or TT (n=9) | 6  5 | 4  1 | 4  0 | 14 (56%)  6 (67%) | 0.44 |
| ***IFNL3/IL28B* rs12979860**  Wild type CC (n=23)  Variant CT or TT (n=11)  Homocygous TT (n=1)  Heterozygous CT (n=10) | 9  2  0  2 | 4  1  0  1 | 4  0  0  0 | 17 (74%)  3 (27%)  0  3 (30%) | 0.03 |
| ***MYD88* rs6853**  Wild type AA (n=25)  Variant GG or AG (n=9)  Homozygous GG (n=1)  Heterozygous AG (n=8) | 9  2  0  2 | 4  1  0  1 | 2  2  1  1 | 15 (60%)  5 (55%)  1 (100%)  4 (50%) | 0.61 |
| ***IRAK2* rs3844283**  Wild type CC (n=18)  Variant GG or CG (n=16)  Homozygous GG (n=1)  Heterozygous CG (n=15) | 7  4  1  3 | 2  3  0  3 | 2  2  0  2 | 11 (61%)  9 (56%)  1 (100%)  8 (53%) | 0.63 |
| ***IRAK4* rs4251513**  Wild type CC (n=10)  Variant GG or CG (n=24)  Homozygous GG (n=12)  Heterozygous CG (n=12) | 3  8  5  3 | 1  4  3  1 | 2  2  1  1 | 6 (60%)  14 (58%)  9 (75%)  5 (42%) | 0.25 |
