# Supplementary material for: Polygenic Innate Immunity Score to Predict the Risk of Cytomegalovirus Infection in CMV D+/R- Transplant Recipients. A Prospective Multicenter Cohort Study
Source: Front Immunol. 2022 Aug 9;13:897912. doi: 10.3389/fimmu.2022.897912 (PMC9397545; doi:10.3389/fimmu.2022.897912)
Supplement: Supplementary file 3 [file Table_3.docx]

**Supplementary Table S3. Minor allele frequencies (MAF) from a reference population (1000 Genomes Project Phase 3) and in the SOT patient cohort. Only information for SNPs of autosomal chromosome location is shown. The *TLR7* SNP was excluded from the analysis since is located at X chromosome. Hardy-Weinberg *p* values were obtained from a chi-squared test.**

| **SNP** | **Alleles**  **Major>minor** | **MAF in reference population** | **MAF in SOT patients** | **Hardy-Weinberg equilibrium *p* value** |
| --- | --- | --- | --- | --- |
| *TLR2* rs5743708 | G>A | 0.024 | 0.022 | 0.972 |
| *TLR3* rs3775296 | C>A | 0.174 | 0.190 | 0.423 |
| *TLR3* rs3775291 | C>T | 0.324 | 0.356 | 0.861 |
| *TLR4* rs4986790 | A>G | 0.057 | 0.086 | 0.407 |
| *TLR4* rs4986791 | C>T | 0.058 | 0.091 | 0.496 |
| *TLR9* rs5743836 | A>G | 0.131 | 0.453 | 0.804 |
| *AIM2* rs855873 | G>A | 0.055 | 0.078 | 0.663 |
| *IFI16* rs6940 | A>T | 0.119 | 0.078 | 0.663 |
| *IL28B* rs12979860 | C>T | 0.309 | 0.259 | 0.993 |
| *MYD88* rs6853 | A>G | 0.131 | 0.155 | 0.854 |
| *IRAK2* rs3844283 | C>G | 0.418 | 0.323 | 0.415 |
| *IRAK4* rs4251513 | C>G | 0.496 | 0.483 | 0.035 |
